# Liquid biopsy for infectious diseases: a focus on microbial cell-free DNA sequencing

**DOI:** 10.7150/thno.45554

**Published:** 2020-04-07

**Authors:** Dongsheng Han, Rui Li, Jiping Shi, Ping Tan, Rui Zhang, Jinming Li

**Affiliations:** 1 National Center for Clinical Laboratories, Beijing Hospital, National Center of Gerontology; Institute of Geriatric Medicine, Chinese Academy of Medical Sciences, P.R. China.; 2 Graduate School, Peking Union Medical College, Chinese Academy of Medical Sciences, Beijing, P.R. China.; 3 Beijing Engineering Research Center of Laboratory Medicine, Beijing Hospital, Beijing, P.R. China.; 4 Peking University Fifth School of Clinical Medicine, National Center for Clinical Laboratories, National Center of Gerontology, Beijing Hospital, Beijing, China.

**Keywords:** microbial cfDNA, cell-free DNA sequencing, metagenomics, next-generation sequencing, microbiology.

## Abstract

Metagenomic next-generation sequencing (mNGS) of microbial cell-free DNA (mcfDNA sequencing) is becoming an attractive diagnostic modality for infectious diseases, allowing broad-range pathogen detection, noninvasive sampling, and rapid diagnosis. At this key juncture in the translation of metagenomics into clinical practice, an integrative perspective is needed to understand the significance of emerging mcfDNA sequencing technology. In this review, we summarized the actual performance of the mcfDNA sequencing tests recently used in health care settings for the diagnosis of a variety of infectious diseases and further focused on the practice considerations (challenges and solutions) for improving the accuracy and clinical relevance of the results produced by this evolving technique. Such knowledge will be helpful for physicians, microbiologists and researchers to understand what is going on in this quickly progressing field of non-invasive pathogen diagnosis by mcfDNA sequencing and promote the routine implementation of this technique in the diagnosis of infectious disease.

## Introduction

More than 1,000 microbes are known to cause human disease, and differential diagnosis of infectious disease is a complex and challenging task in the clinic. In some infectious diseases, such as encephalitis and bloodstream infection, over 50% of cases cannot obtain a clear pathogenic diagnosis 1-3. Microbiological culture-based methods (e.g., microscopy, special staining, serology, etc.) are the preferred tests used for the identification of common pathogens, but the turnaround time-to-results period is long (≥ 48 h), and many pathogens are difficult or impossible to culture. Multiplex polymerase chain reaction (PCR) tests are rapid but typically capture a small number of etiological agents and need a presumptive diagnosis before a test is chosen 4. Mass spectrometric (MS) techniques have attracted much attention in the identification of clinical pathogens (bacteria, fungi, and viruses), but they still need organisms isolated in pure culture 5. Recently, unbiased metagenomic next-generation sequencing (mNGS), through detecting microbial nucleic acids in a variety of specimens to detect potential pathogens in culture-negative patients, has demonstrated to be a very promising microbial identification technology 6-9. For many tests, however, invasive sampling (e.g., cerebrospinal fluid (CSF), tissues, bronchoalveolar lavage, etc.) cannot be avoided. Once faced with life-threatening infectious patients who cannot withstand invasive procedures, these techniques are powerless. Therefore, the ability to identify pathogens causing infection throughout the body from noninvasive samples (such as peripheral venous blood, urine, etc.) remains an unmet clinical requirement.

Liquid biopsy based on circulating cell-free DNA (cfDNA) provides a new prospect for the diagnosis and treatment of clinical infectious diseases. cfDNA molecules in circulation originate from dying human cells as well as from colonizing or invasive microbes that release their nucleic acids into the blood as they break down 10. Human-derived cfDNA has evolved into an indispensable biomarker in clinical practice for rapid and noninvasive diagnosis in prenatal screening, transplantation and oncology 11-15. Although early studies did not focus on cfDNA of microbial origin (hereinafter referred to as mcfDNA) because of the limited understanding of these small molecules 16, it is clear that the development of circulating cfDNA-based tests for infectious diseases has recently been gaining traction in clinical practice. An increasing number of studies have demonstrated that mcfDNA detection offers the potential to reliably identify a wide variety of infections, such as invasive fungal infection 17, tuberculosis 16 and sepsis 18.

Early detection of mcfDNA in body fluids used mainly various PCR methods (e.g., conventional PCR, nested PCR, Real-time PCR, droplet digital PCR (ddPCR), etc.) 19,20. Recently, Liao et al. constructed a ZIKV liquid biopsy system based on a dendritic Ru(bpy)3 2+-polymer-amplified electro-chemiluminescence (ECL) strategy, with which, Zika Virus RNA could be identified using even a drop of blood 21. These tests are simple, rapid and sensitive but limited to a narrow spectrum of the most common pathogens. mcfDNA-based next-generation sequencing (mcfDNA sequencing) is an emerging hypothesis-free test that detects mcfDNA shed into noninvasive samples (such as peripheral venous blood, urine, etc.) from sites of infection. On the basis of high-throughput sequencing, it offers the potential to identify a wide range of infections throughout the body in a single sequencing run, including cases where there has been antibiotic pretreatment prior to cultures and in those with fastidious, difficult-to-culture organisms 22. In 2019, Blauwkamp and colleagues validated a plasma mcfDNA sequencing assay, described as the Karius test, and proposed that this technique is now clinically relevant and actionable and offers distinct advantages over traditional diagnostic methods in feasibility, invasive procedure avoidance, cost effectiveness, and clinical outcomes 4.

In this review, we focus primarily on the recent implementation of mcfDNA sequencing tests in the clinical context for the diagnosis and evaluation of infectious diseases and further discuss the key factors and possible solutions that affect the stability and accuracy of their results in the preanalytical (sample collection handling and processing), analytical (cfDNA isolation, library preparation, sequencing and bioinformatics analysis) and postanalytical (results interpretation and reporting) phases. This knowledge will help readers obtain a comprehensive understanding of this emerging and evolving diagnostic technology for infectious diseases.

### Overview of the Biological Characteristics of mcfDNA

The detected concentration of plasma cfDNA in healthy individuals varies greatly, generally within the range of 0-100 ng per milliliter of plasma, sometimes exceeding 1500 ng per milliliter 23. Human DNA accounts for the vast majority (>90% or even >99%), while mcfDNA accounts for only a small fraction (0.08%-4.85% from bacteria, 0.00%-0.01% from fungi, and 0.00%-0.16% from viruses/phages) 24. Elevated levels can be observed in a variety of pathological conditions, including infection, sepsis, trauma, and autoimmune diseases 4,25,26.

**Figure 1:**
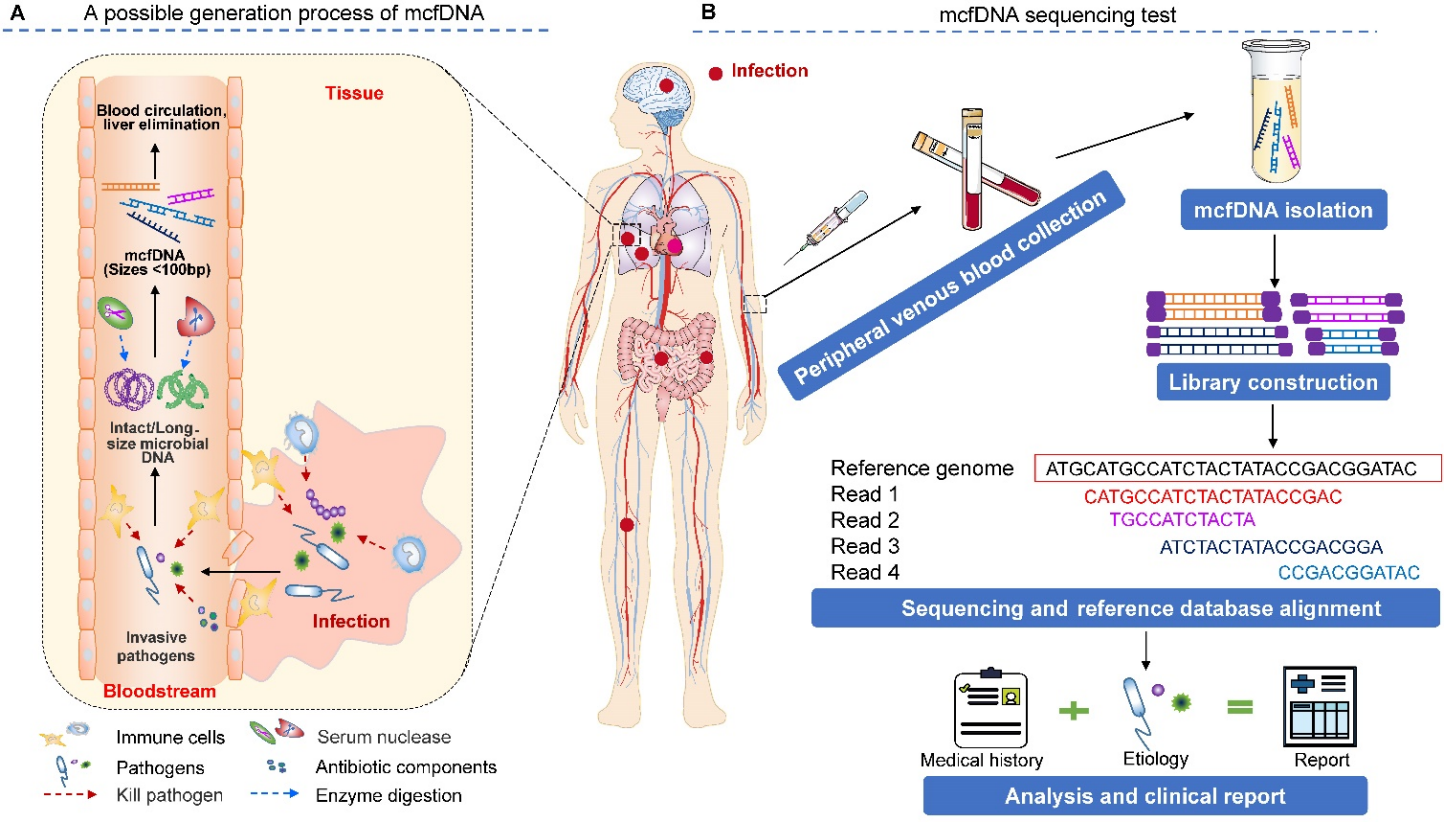
The principle and procedure of the mcfDNA sequencing test for identifying pathogens causing invasive infection. **(A)** A schematic drawing of the possible origin, release, and degradation of mcfDNA. Infections at diverse body sites can produce circulating pathogen cfDNA. However, existing studies have not provided sufficient evidence to show the true contribution of various immune cells and biological components in the process of mcfDNA generation, which requires additional research to explore. **(B)** A workflow of the mcfDNA sequencing test. The figure is adapted with permission from 87, copyright © 2019, Springer Nature.

The source of mcfDNA in circulation is an intriguing question (Figure 1A). Traditionally, human blood is considered sterile. The detected mcfDNA may have two sources, including (1) microbial (bacterial, virus or bacteriophage) translocation 27-30, which refers to the process by which the microbial cells that belong to the human microbiome or their components (such as lipopolysaccharide (LPS), peptidoglycan, DNA, etc.) enter the circulation through the epithelial mucosa of organs that communicate with the external environment (e.g., the gastrointestinal tract, oral cavity, reproductive tract, etc.). For example, in the gastrointestinal tract, this phenomenon occurs when intestinal microorganisms overgrow, intestinal mucosal barrier permeability increases, and host immune defense becomes defective 29. Additionally, (2) when the tissue mucosa is damaged by local infection (e.g., oral, lung, and skin infections) or physical damage (e.g., invasive surgery or accidental injury), invasive pathogens may opportunistically enter the bloodstream, causing bacteremia or viremia in severe cases 27. These invading microbes can be killed and disintegrated by antiinfective drugs and the body's immune response (for example, neutrophils can eliminate microbes by phagocytosis, generating reactive oxygen species (ROS), releasing microbicidal molecules from granules (degranulation) and forming neutrophil extracellular traps (NETs) 31), resulting in the release of microbial nucleic acids 32-34. Once in the circulation, microbial nucleic acids are degraded via circulating exonucleases (enzymes whose involvement in this process is not well understood; DNase II may be involved 35) and finally form small DNA fragments, i.e., mcfDNA. Owing mostly to the lack of protection from histone octamers and large, persistent transcription factors 36, the size distribution of mcfDNA is consistently shorter than that of human nuclear DNA in plasma. Zhang et al. observed that the size profile of mcfDNA in plasma does not show a 166 bp major peak or smaller peaks occurring at a periodicity of 10 bp. mcfDNA is approximately 40-100 bp, with a GC content of 43.5% 37. Microbial sequencing methods are used to diagnose possible infections by capturing and identifying this highly fragmented mcfDNA in the circulatory system. Further studies have shown that the half-life (a few minutes) of mcfDNA is shorter than that (10-15 min) of protein-bound (nucleosomal) DNA 23,25. Liver elimination is the main mechanism of circulating DNA clearance from plasma 23. With a favorable treatment outcome, the plasma cfDNA may remain stable for the first week and be completely eliminated within a 2-3-week period 17,38.

### Entering the Clinic: The Diagnostic Potential of mcfDNA Sequencing for Infectious Diseases

The mcfDNA sequencing test has been applied to diagnose a wide range of clinical infectious diseases such as bloodstream infection, pulmonary and extrapulmonary tuberculosis (TB), invasive fungal/parasitic infection 17, endocarditis, complicated pneumonia 4, urinary tract infection 39 and secondary infection after solid organ transplantation 40. Studies reported that the whole process from specimen preparation in the laboratory to results could be accomplished within a clinically actionable timeframe (2-3 days), providing clinically useful information to ensure the effective treatment of patients 4,18,25,41. However, it should be noted that since most of the mNGS tests are currently available only in third-party laboratories (such as the validated Karius test), the process from specimen collection in health care settings to transportation to the laboratory may delay the final diagnosis of the disease. For example, Farnaes et al showed that, plus sampling and shipping time, the average time to mcfDNA sequencing result was 98.1 h (range 48-245.3 h) 42. At present, several factors limit the establishment of metegenomic workflow in the routine microbiology laboratory. For example, (1) the current sequencing platforms integrated into mcfDNA sequencing pipelines are mainly Illumina sequencers (HiSeq or NextSeq) 4,43,44. The purchase of these equipment tends to be more than $500,000 and the cost of reagents for the following analytical and clinical validation will exceed $100,000 45. These costs are higher than that of any other test currently established in clinical microbiology laboratory; (2) From the perspective of patients, in the absence of health insurance support (i.e., reimbursement is unlikely), they may not give priority to this technology, because the average cost of each test is more than $2000 45-47; (3) The operation procedure is complex, and there are many factors may affecting the accuracy of the results to be considered from the preanalytical phase to the postanalytical phase (which will be discussed in the following sections) (Table 1); and (4) the requirement for special bioinformatic education and skills 48. With the possible cost decreasing, technology optimization and the establishment of metagenomic platforms in routine laboratories in the future 45, the turnaround time can be further shortened.

Comparisons showed that the mcfDNA sequencing test yielded a higher positive rate than culture and other conventional microbiological methods (Table 2). Although additional well-designed prospective studies with sufficient power and specimen size are needed, several studies with tens to hundreds of subjects initially assessed the sensitivity (70.0%-92.9%) and specificity (62.7%-88.2%) of mcfDNA sequencing for pathogen identification using the results of conventional methods and/or clinical judgment as reference standards (Table 2). The positive predictive value and negative predictive value for bacteremia were calculated as 53.3% and 95.2%, respectively, in an mcfDNA sequencing analysis of 78 plasma specimens from ICU patients 18. Most importantly, mcfDNA sequencing is expected to become a reliable screening test for predicting clinical infections. Through a prospective pilot cohort study of mcfDNA sequencing in blood samples from 47 relapsed pediatric cancer patients with impending bloodstream infection (BSI), Goggin et al. provided the evidence that plasma mcfDNA sequencing test could predict BSI 3 days before onset in approximately 75% of patients with an overall specificity of 82% (95%CI, 66%-91%), potentially guiding preemptive therapy 26.

A multicenter retrospective study showed that although the advantage of the currently used cfDNA sequencing test as a first-line tool in the diagnosis of common infectious cases is not obvious, it is of great significance in the establishment of a new diagnosis, earlier diagnosis than that provided by conventional methods and escalation/de-escalation of therapy as used in routine practice 49. More importantly, the characteristics of noninvasive sampling provide great convenience for clinical practice. mcfDNA assays in conjunction with conventional diagnostic techniques may significantly increase diagnostic yield and facilitate antibiotic selection in infections such as severe CAP and sepsis 4,50. Further studies are needed to determine the optimal patient populations, define the complementary role of mcfDNA sequencing to other diagnostic methods of infectious diseases, and identify how best to integrate mcfDNA sequencing into the current clinical microbiological identification system 49.

### Success Stories and Attempts at Implementation of mcfDNA Sequencing

#### Bloodstream Infections

Bloodstream infection remains one of the major challenges in the clinic, leading to sepsis or even septic shock in many cases 51. Due to the lack of rapid diagnostic approaches to identify causative pathogens, mortality rates of sepsis are still unacceptably high. In 2016, Grumaz et al reported a complete diagnostic mcfDNA sequencing workflow that was capable of identifying the pathogens causing sepsis from plasma specimens within 30 h from sampling to result reporting 25. They also demonstrated that the concentrations of plasma mcfDNA in septic patients increased significantly compared with those in healthy volunteers (average classified reads for microbes: 9.82% vs. 3.50%). Other proof-of-concept studies showed that compared with conventional culture methods, the mcfDNA sequencing test significantly improved the pathogen detection rate (approximately 20%-30%) in sepsis specimens (Table 1), providing useful information for establishing rational antibiotic treatment plans and revealing the pathogen profiles of sepsis patients 4,18. To date, several attempts have been made to rapidly and accurately diagnose bloodstream infections by using different mcfDNA sequencing workflows. For example,* Propionibacterium acnes* (*P. acnes*), a common bacterium of the skin flora, was identified as the causative agent in a boy with *juvenile myelomonocytic* leukemia presenting with signs of infection while traditional clinical diagnostic tests failed to detect any pathogenic agent. This result was confirmed by qPCR assay and effective antimicrobial treatment of *P. acnes* 41. Monica et al described 3 allogeneic hematopoietic stem cell transplant patients for whom plasma mcfDNA sequencing could have facilitated prompt identification of an uncommon presentation of *Chlamydia trachomatis* (a month earlier than standard microbiology) and indicated persistent MRSA infection before microbiologic diagnosis of recurrent bacteremia and metastatic infection 52. In another report, mcfDNA sequencing was successfully used to diagnose a *Capnocytophaga canimorsus* infection in an asplenic patient presenting with culture-negative sepsis, showing its promise in identifying fastidious pathogens 22.

In 2019, Blauwkamp et al. described the first commercial quantitative plasma mcfDNA sequencing test (the Karius test) 4. It presented a sensitivity of 92.9% and a specificity of 62.7% in comparison to a composite reference standard (including culture, serology and nucleic acid testing results and clinical adjudication) when testing the plasma specimens from a cohort of 350 suspected sepsis patients. Moreover, the assay was capable of detecting a probable cause of sepsis in 48.6% of patients compared with 18.1% identified by blood culture and 37.9% identified by all microbiological testing combined (i.e. cultures, serology, nucleic acid testing) (Table 2). Even more valuable, the authors found that the mcfDNA sequencing test performed much better than blood culture (pathogen detection rate: 47.9% vs 19.6%) in analyzing specimens from subjects who had received antimicrobial therapy within two weeks preceding presentation.

Altogether, increasing evidence supports the notion that the mcfDNA sequencing method is a valuable tool in the early diagnosis of blood infections caused by uncommon/unexpected pathogens and in situations of atypical clinical presentations, potentially allowing for early targeted therapy to the improve clinical outcomes and decrease the antimicrobial resistance and drug toxicity of bloodstream infections.

#### Tuberculosis (TB)

TB is a good example of an infectious disease for which the cfDNA sequencing test would be especially promising. Clinical recognition of TB is hampered by its long latency and nonspecific presenting symptoms. Etiological diagnosis is typically delayed when reliant solely on acid-fast bacillus (AFB) culture, and invasive biopsies are often necessary to cultivate the pathogen from deep-seated infections 53. To make an early diagnosis of tuberculosis, researchers have established several targeted *Mycobacterium tuberculosis* cfDNA assays (PCR-based methods) to determine the presence of infection by detecting cfDNA in blood and urine specimens, demonstrating that mcfDNA could be an attractive biomarker for TB detection and treatment monitoring 16. More recently, the performance of the mcfDNA sequencing test was evaluated in patients with tuberculosis infection. For example, Nomura et al described the successful application of a plasma mcfDNA sequencing test for direct detection in a series of cases of invasive* Mycobacterium chimaera* infection, providing accurate noninvasive microbiologic confirmation of this fastidious organism more than one month faster than standard AFB culture. Even if the patient had received antibiotic pretreatment, a pathogen cfDNA signal could also be detected from plasma 53. Similarly, other successful applications in diseases such as opportunistic *Mycobacterium avium or Mycobacterium tuberculosis* infections in HIV/AIDS patients 54 and aneurysms infected by *Mycobacterium bovis* due to Bacille Calmette-Guérin (BCG) instillation 55 demonstrate that this new approach is a promising, less-invasive diagnostic and monitoring tool for TB.

#### Invasive Fungal Infections (IFDs)

The widespread use of immunosuppressive regimens and a rise in antifungal-resistant organisms has led to invasive fungal infections (IFDs), which remain a major cause of morbidity and mortality in immunocompromised patients 56. Given the wide diversity of pathogenic fungi, there is a critical need for rapid, noninvasive, species-level identification of these invasive infections to help guide specific antifungal therapy. In 2018, Hong et al first reported the use of plasma cfDNA sequencing in patients with proven IFD and was able to detect the same fungus identified from biopsy tissue 17. This study demonstrated that pathogen cfDNA from deep-seated infections caused by difficult-to-culture molds, such as* Aspergillus*, *Rhizomucor*, and *Scedosporium* species, can be less invasively identified by directly sequencing plasma specimens, potentially providing a more rapid diagnosis and obviating the need for invasive biopsies. Plasma mNGS also identified the invasive fungal pathogen *Histoplasma capsulatum* in a pneumonia patient with disseminated disease 57, and diagnosed the co-infection with two fungal pathogens (*Cunninghamella bertholletiae* and *Aspergillus lentulus*) producing invasive disease in a 62-year-old hematopoietic stem cell transplant recipient with graft-versus-host disease (GVHD) 58. In another report about a cluster of cases of pneumocystis pneumonia, Zhang et al. successfully identified *Pneumocystis jirovecii using* mcfDNA sequencing in peripheral blood specimens from 3 pneumocystis pneumonia (PCP) patients who could not withstand bronchoscopy examination or declined invasive operation 59. In a recent pilot study, the authors confirmed that mcfDNA sequencing could accurately and noninvasively identify fungal pathogens in 5 of 7 pediatric patients with new IFD 60. In this study, the causal fungal pathogens (*Aspergillus fumigatus*, *Candida albicans* and* Rhizopus delemar*) identified by mcfDNA sequencing were consistent with the results obtained by lung biopsy, skin biopsy, blood or pancreatic pseudocyst cultures*,* demonstrating the ability of an mcfDNA test to detect fungal pathogens at the species level from various infection sites. Overall, the existing data supports the emerging promise of plasma mcfDNA sequencing to address the unmet clinical need in IFD diagnosis in at-risk patients, guiding treatment decisions and limiting excessive empiric antifungal use.

#### Infections in Organ Transplant Patients

Clinically, patients undergoing organ transplants need to use immunosuppressants for a long time to reduce the risk of rejection, but this therapy increases the risk of infection. Diagnosis of concurrent infection in organ transplant patients is challenging given that symptoms of infection are often diminished after immunosuppression and that many diagnostic tests are sensitive to only one or a few pathogens. Several emerging applications of cfDNA sequencing in a variety of transplant settings (e.g., kidney 61, allogeneic hematopoietic stem cell 41,52 and lung 62,63) are rapidly filling a critical medical need for more informative, noninvasive assays for acute rejection, infection, and immunosuppression.

#### Other Cases of Interest

The mcfDNA sequencing test has also been implemented in the diagnosis of other infectious diseases. For example, Kondo et al performed plasma mcfDNA sequencing to facilitate rapid diagnosis (within 48 h) and genotyping of *Coxiella burnetii* in a patient with culture-negative endocarditis of a prosthetic pulmonary valve, enabling early targeted treatment prior to valve replacement surgery 64. Langelier et al. showed that plasma cfDNA sequencing identified one or more clinically-confirmed pneumonia pathogens in 13/18 (72%) bacterial pneumonia cases 57. In the future, plasma cfDNA sequencing might be leveraged for the simultaneous identification of early cancer and diagnosis of infection in immunocompromised patients 65. Urine cfDNA sequencing analyses permit the identification of a broader spectrum of bacterial species in infections of the urinary tract 39. In addition to being a superior tool for the identification of infections, mcfDNA analysis is highly informative in monitoring changes in the microbiome architecture 40,66 and assessing the severity of diseases when integrating the host injury response to infection 61. However, all of these findings are limited to individual studies, and large studies are needed to assess their clinical availability.

### Can mcfDNA Sequencing be Used to Identify Parasite Infections?

Several studies have reported that mcfDNA molecules could be used as diagnostic markers for human parasitic infections. With the help of conventional PCR-based methods, cfDNA belonging to parasites such as *Entamoeba histocytica* 67, *Plasmodium spp*. 68 and *Schistosomiasis mansoni* 69 have been detected in serum, and *Leishmania*-derived cfDNA has been found in urine 70. We feel that the mcfDNA sequencing approach will be more sensitive than PCR methods because the mcfDNA sequencing test (1) can theoretically identify all the DNA fragments of pathogens except RNA viruses in blood, including parasite DNA, and (2) is more sensitive than PCR methods in detecting short random cfDNA fragments with sizes (<100 bp) close to or smaller than the PCR amplicon length. However, at the time of this writing, there have been no reports on the application of cfDNA sequencing in the diagnosis of parasitic infectious diseases. This possibility requires systematic evaluation by further studies.

### Is mcfDNA Sequencing Feasible for Monitoring Antibiotic Resistance?

To maximize the impact on patient management, it is equally important to identify clinically relevant antibiotic resistance genes. Based on NGS-based diagnosis, several studies have successfully identified genes conferring antibiotic resistance in plasma cfDNA 25,57. For example, Grumaz et al unambiguously identified reads that exactly matched (100%) the vancomycin resistance genes *vanB* and *vanSB* from a liver transplantation patient. Moreover, using their approach, they identified vancomycin-resistant Enterococcus (VRE) as the infectious organism in a patient and methicillin-resistant *S. aureus* carrying the *mecA* gene in other patient plasma specimens 25. However, these reports are the exception rather than the rule. Indeed, in complex clinical situations, predicting the resistance phenotypes of the detected isolates through detecting resistance genes via metagenomic sequencing is not always reliable. A recent mNGS assay using a Nanopore sequencing workflow illustrated the complexity of the detected resistance genes 71. They found 183 resistance genes in 41 clinical specimens, but surprisingly, only 24 (13.11%) matched the resistance observed by antimicrobial susceptibility testing (AST). Among the other detected genes, some were only partially related to the phenotype of the isolates cultured, several were completely contradictory to the phenotypic resistances, and nearly 1/3 of the detected genes (56/183) were derived from the normal or colonizing respiratory flora. Such a situation makes it very complicated to interpret the clinical significance of each gene detected by sequencing methods.

Given that cfDNA sequencing detects highly fragmented, irregular DNA sequences, it is difficult to assemble these sequences into complete resistance genes. When using these incomplete sequences to match the reference gene sequences, false positives are likely to occur 72. Additionally, the imperfection of antibiotic resistance databases is another issue that hampers the accurate identification of antibiotic resistance 25. We urgently need such databases that have a low false negative/positive rate for known antibiotic resistance gene (ARG) prediction, can predict genotype and phenotype relationships and can be continuously updated to include newly discovered genes in a timely manner 72-74. Furthermore, from a diagnostic perspective, limits of detection need to be established to have sufficient coverage for the respective species with the capacity to detect ARGs or in complex metagenomes 75. Similarly, the specificity and sensitivity of the developed method for resistance gene detection should be determined as well, but it will be a very difficult job, as this determination would have required isolating and sequencing all bacteria (pathogens and commensals) present 71. Another fact to consider is that even confirming the presence of a resistance gene does not guarantee that it is expressed or confers antibiotic resistance on its host 72. Last, we also need to understand that resistance gene detection often requires a higher sequencing depth than bacterial identification (10-100-fold) 25. The higher the sequencing depth is, the higher the cost.

Thus, even if the genotypic inference of antimicrobial susceptibility from sequencing data continues to improve, metagenomics sequencing of fragmented cfDNA alone without directed amplification of relevant loci is merely a substitute for other molecular speciation methods and cannot routinely replace phenotypic testing of clinical isolates 43.

### Important Practice Considerations

Similar to other routine tests in the clinical laboratory, the process of mcfDNA sequencing can be divided into the preanalytical (sample collection handling and processing), analytical (cfDNA extraction, library preparation, sequencing and bioinformatics analysis) and postanalytical (results interpretation and reporting) phases (Figure 1B). At each phase, to maximize accuracy and clinical relevance, multiple factors that may induce analytical biases should be taken into account when implementing a clinical mcfDNA sequencing pipeline for the diagnosis of infections.

#### Preanalytical Considerations

Care in the preanalytical handling of clinical specimens is vital for the successful implementation of cfDNA analysis in clinical practice. However, no standard operating procedure (SOP) for sampling has yet been published despite increasing clinical studies on microbial cfDNA sequencing. By carrying out experiments using blood and urine specimens spiked with small DNA fragments from four pathogens (*Mycobacterium tuberculosis*, *Salmonella enterica*, *Aspergillus fumigatus* and *Epstein-Barr virus* (EBV)), Murugesan et al. found feasible and inexpensive preanalytical steps for the recovery of pathogen cfDNA from blood and urine, including (1) sampling a large volume (4 ml) of plasma (collected with K2-EDTA blood collection tubes) and whole urine preserved with 25 mM EDTA, (2) single-spin low-speed plasma separation (500 × g for 10 min at room temperature) rather than a double-spin separation, and (3) a processing delay within 24 h 76. They also described that freezing and thawing of plasma or urine specimens after storage at -80 °C for up to 6 months did not influence the abundance of pathogen cfDNA. In the commercial Karius test, qualified specimens need to meet the following criteria: (1) whole blood should be collected in a K2-EDTA or BD Vacutainer PPT tube (Becton Dickinson, Franklin Lakes, NJ); (2) a minimum of 1.2 ml of plasma is required to be separated from the blood; (3) plasma separation must carried out within 6 hours after blood draw; and (4) specimens should be delivered to the laboratory either fresh (within 4 days of the blood draw) or frozen 4,49. Other studies highlighted that cfDNA extracts could be stored at -20°C or -80°C and should not undergo >3 freeze-thaw cycles to avoid further degradation 77,78.

Another important concern is nucleic acid contamination, which is derived mainly from human DNA produced by human leukocyte lysis and exogenous microbial DNA introduced during specimen processing. As mentioned above, the level of pathogen cfDNA in specimens is generally trace. Human DNA contamination will easily reduce the detection sensitivity of pathogen cfDNA (the false negative problem). Exogenous microbial DNA contamination will increase the complexity of interpretation of results (the false positive problem). In February 2019, based on a large number of previous studies on delineating preanalytical variables, Meddeb et al. proposed general guidelines for analyzing cfDNA 77. We believe the following measures mentioned in the guidelines on how to effectively avoid or reduce leukocyte release are equally applicable in microbial cfDNA analysis despite further validation being necessary. (1) When drawing blood, large gauge needles (<21 gauge) are recommended to keep blood cells as intact as possible. (2) Plasma separation should be performed as early as possible, with a delay not exceeding 4 h, using K2-EDTA tubes. If plasma isolation must be delayed, blood can be stored in K2-EDTA tubes at 4 °C for up to at least 24 hours. This recommendation is similar to the finding by Murugesan et al. 76. (3) To avoid hemolysis, blood tubes need to be gently inverted 8 to 10 times but not shaken and should be transported in an upright position. Finally, (4) blood clotting must be carefully checked for, which may also lead to blood cell disruption.

The sources of exogenous microbial DNA contamination vary depending on sampling sites (e.g., skin), laboratory surfaces, consumables and reagents used for cfDNA analysis 65. Even miniscule amounts of exogenous DNA can complicate the analysis and interpretation of results. There are numerous options to minimize the effects of DNA contamination in mcfDNA sequencing analysis. For example, during sampling and processing, experimenters should wear protective clothing and equipment (i.e., lab coats, face masks, hairnets, sleeves, and clean disposable gloves) to cover all exposed skin, if possible, to reduce the introduction of contaminants into the specimens 79,80. As many procedures as possible, such as the preparation of consumables and reagents, plasma separation and aliquoting, etc., need to be completed in a cleaned, isolated working environment. Highly trained personnel are especially required in preanalytical steps to avoid errors and putative cross-​contamination. In addition, necessary negative controls (a sampling blank control, DNA extraction blank control, and no-template amplification control), reagent assessments and periodic wiping tests are important ways to monitor laboratory and specimen cross-contamination 80.

#### Analytical Considerations

According to our practical experience, the final concentration of cfDNA extracted from 300 µl of patient plasma is often no more than 1 ng/µl (in an elution volume of 50 µl). To maximize the recovery of cfDNA, the development of efficient cfDNA extraction methods is critically important. Recently, Cook et al. demonstrated that the extraction yields of cfDNA extraction kits were extremely variable across the variety of methods/instruments used and fragment concentrations in specimens, with the 50- and 100-bp fragment sizes (that correspond to the sizes of the mcfDNA in circulation) showing especially inconsistent quantitative results and poor yields of less than 20% of the expected fragment concentrations 81. This finding may imply that many methods do not have a satisfactory ability to extract small fragment nucleic acids, such as mcfDNA. Further studies are necessary to validate the performance of the existing mcfDNA extraction kits, develop new methods/kits and determine the clinical utility of improved methods/kits for infectious disease diagnostics.

In other respects, researchers have developed several methods from different theoretical perspectives in an attempt to increase the detection sensitivity and specificity of mcfDNA sequencing in specimens. For example, using the prior finding that mcfDNA is more fragmented than human cfDNA in plasma, which has a predominant peak at 166 bp, Murtaza and colleagues developed a size-selection assay for mcfDNA sequencing 82. Briefly, they selected only a subset of the extracted cfDNA with a size below 160 bp, 150 or 140 bp to perform whole genome sequencing and then assigned the sequencing data to a microbial database to determine the possible pathogen in a plasma specimen. The validation based on 82 plasma specimens from 30 patients showed that there was a median 24.7-fold enrichment in the fraction of sequencing reads classified as bacterial in the size-selection method compared to that without a size-selection process. Burnham et al. developed a single-stranded DNA (ssDNA) library preparation method for cfDNA sequencing, which was demonstrated to be more sensitive in recovering ultrashort and degraded bacterial and viral cfDNA in plasma than a double-stranded DNA (dsDNA) library preparation method 37. Subsequent data showed that this ssDNA library preparation method could provide a mean 71-fold increase in the relative genomic coverage of microbial species and could detect many species that were not observed in the dsDNA library preparation assays 36. In addition, the sensitivity of mcfDNA sequencing in plasma may also be boosted by increasing the sequencing depth to obtain additional sequencing data, but this approach will increase the analysis time and test cost. Recently, Burnham et al. reported a bioinformatics tool named low biomass background correction (LBBC) for separating the signal from the noise (i.e., alignment noise, annotation errors in reference genomes, and environmental contamination) in metagenomic cfDNA sequencing 83. This tool enabled the cfDNA sequencing assay to identify urinary tract infection with enhanced specificity while minimally affecting its sensitivity.

As with any clinical test, the implementation of control specimens is necessary for real-time monitoring of biases and errors in next-generation sequencing tests 84,85. However, no commercial well-characterized positive controls or reference materials are currently available for mcfDNA sequencing tests, which is also a problem for other mNGS analyses 65. For the mcfDNA sequencing test, prior to the availability of commercial reference materials, residual clinical specimens that have been confirmed by previous cfDNA sequencing tests or cfDNA PCR tests with or without DNA fragments of interesting pathogens could be used as control specimens. The key point is setting reasonable conditions for the control specimen aliquoting and storage to ensure that these specimens are not contaminated and that target mcfDNA is not degraded. In addition, the development of in-house reference materials for different testing purposes should be encouraged. For example, to perform analytical validation of the Karius test workflow, Blauwkamp et al. created sheared genomic DNA (gDNA) specimens of 14 representative pathogens as cfDNA control specimens 4. These sheared gDNAs were produced by enzymatic shearing of each reference pathogen genome, and their fragment lengths were in the range of 60-90 bp, which corresponds to the distribution of microbial cfDNA detected in clinical specimens.

#### Postanalytical Considerations

Since plasma mcfDNA can stem from the site of infection or colonization 86, it is challenging to distinguish causative pathogens from others (normal microbes and environmental contaminants). Although the validated Karius test reports quantitative results, there are no clear cutoffs that differentiate infection from colonization or contaminants 4,49. Therefore, care must be taken when interpreting the mcfDNA sequencing test results, considering both the pathogen(s) identified and the clinical manifestation, especially for immunocompromised patients 87. To obtain a reasonable and accurate interpretation of the results, the following approaches will be beneficial. (1) Normal clinical noninvasive specimens (e.g., blood, urine, etc.) should be sequenced to establish and maintain a benchmark database showing the types and amounts of microbial cfDNA in healthy or noninfected populations. Microbes in this database are either not reported or will require higher thresholds (quantitative results of cfDNA fragments) for reporting if they are clinically significant microbes. This strategy has been used in the interpretation of the results of mNGS assays for other specimen types (e.g., CSF, intraocular body fluid, etc.) 88,89. (2) Statistical models to improve the ability to automatically identify real pathogens should be developed. For example, Grumaz et al. created a sepsis-indicating quantifier (SIQ) score to discriminate signal reads from noise caused by contaminant or commensal species in sepsis patient blood specimens 25,90. Langelier et al. utilized a Bayesian scoring metric and the calculated Z-scores for pneumonia pathogen assessment and background contaminant correction from plasma cfDNA 57. Using a developed Random Forest classifier and a bacterial co-occurrence network, Chen et al. rapidly identified pathogenic bacteria and diagnosed sepsis from cfDNA sequencing data 91. (3) Before reporting the final positive results to the clinic, other methods (e.g., culture, serological testing, PCR, Sanger sequencing, etc.) should be considered to verify the presence of the pathogen or infection 60**.** (4) Drawing on the successful experience of the CSF mNGS test 92,93, it is necessary to build a multidisciplinary team to evaluate the clinical significance of the findings. Especially for challenging cases or confusing results, microbiologists, clinicians, and bioinformatics technicians in this team can discuss and make the most beneficial decisions for patients in the context of all data available 94.

A final concern with cfDNA sequencing tests is the lack of detection of RNA virus pathogens 4, including many important RNA viruses associated with human infections, such as human immunodeficiency virus (HIV), Zika, hepatitis C virus (HCV), respiratory syncytial virus (RSV), enteroviruses and norovirus. If a disease is clinically suspected to be caused by RNA virus infection, alternative approaches such as serological tests, qPCR, RNA-seq and other unbiased metagenomic approaches (e.g., mNGS analyses of CSF, respiratory specimens, tissues, etc.) could be considered for differential diagnosis. It is worth noting that Pan et al. demonstrated that asymptomatic viral infections that occurred during pregnancy could be detected using a plasma cell-free RNA (cfRNA) sequencing test 95. If this technique is used more widely, it would be a useful complement to noninvasive methods for detecting infectious diseases.

As a metagenomic shotgun sequencing approach, the cfDNA sequencing method has the same issues as other mNGS sequencing methods based on the whole microbial genomes in clinical samples, from library preparation to result analysis (Table 1). These issues were described in detail in excellent previously published reviews 43,65,80,96.

## Conclusion

It is very clear that the mcfDNA sequencing test for infection diagnosis is gaining traction and is starting to be clinically applied. Although this new technique has limitations and is not routinely implemented in most laboratories, it provides an additional useful diagnostic strategy for clinical infection, as a noninvasive detection technique. To promote wider application of this technique in clinical routine diagnosis, there is an urgent need to carry out research on the following aspects. (1) Comparison with other microbiological methods to determine the advantages of mcfDNA sequencing in various infectious diseases and to define the complementary role of mcfDNA sequencing to conventional microbiological methods should be performed. (2) The development, optimization and analytical and clinical validation of additional mcfDNA sequencing platforms/pipelines should be completed. (3) A variety of universal reference materials should be developed for different clinical contexts and to promote the establishment of a quality assurance system. (4) Multicenter prospective cohort studies should be conducted to show the real-world clinical impact of mcfDNA sequencing for the noninvasive diagnosis of infections and to determine which patient populations are most likely to benefit from this test.

Overall, we believe that with the increasing successful applications in diagnosing infectious diseases, improved methodologies and reduced costs, mcfDNA sequencing tests can be adopted in an increasing number of laboratories in the foreseeable future, resulting in improved patient management, patient outcomes and antimicrobial stewardship.

## Figures and Tables

**Table 1:** Considerations when implementing a mcfDNA sequencing test in clinical practice.

CONSIDERATION OF TECHNICAL PROCESS		
STEP 1. SAMPLE COLLECTION	•Evaluate the best time to collect samples during the course of an illness, because of the rapid clearance of mcfDNA in the bloodstream. •Specimen transport and preservation. •Nucleic acid contamination (environmental microbes, human leukocyte cell).	60
STEP 2. MCFDNA ISOLATION	•Reagents (e.g., elution buffers, nucleic acid extraction kits and enzymes) contaminated by environmental microbial DNA during production. •Extraction efficiency of cfDNA isolation kits. •Reference materials (well-characterized control samples).	43
STEP 3. LIBRARY PREPARATION	•Contaminations. •Long hands-on time (4-6h). •Library quantification and normalization (e.g., low library complexity, flow cell overloading/underloading and index hopping).	43,65,97
STEP 4. SEQUENCING	•Cost (Instruments>$500,000, regents>$100,000). •Sequencing run times. •Minimum read depths. •Sequencing error (quality).	43,45,80,98
STEP 5.BIOINFORMATIC ANALYSES	•User-friendly computational pipeline. •Concordances among different bioinformatics tools. •Software validation. •Pathogen database quality.	65,97,99,100
STEP 6. INTERPRETATION AND REPORTING	•Define a reasonable standard for interpreting results. •Build a multidisciplinary team to evaluate the confusing results. •Miss RNA viruses. •Protect patient privacy. •Data storage (method, location, duration and security measures).	65,80
**CONSIDERATION OF PATIENTS' NEEDS**		
PATIENTS' NEEDS	•Informed consent. •The test reliability. •Lower cost (relatively expensive ($2,000 per test)). •Shorten turnaround time (remains too long for the diagnosis of serious acute infection). •Reimbursement. •Personal privacy protection.	47,101

**Table 2:** The performance of mcfDNA sequencing versus initial blood culture and other microbiological methods reported by previous studies.

ID	Diseases	Case/ Specimen(n)	Pathogen detection rate			Sensitivity %	Specificity %	Reference
			Culture (%)	Other methods (%)	mcfDNA sequencing (%)			
1	Spesis	78	12.8(10/78)	-	30.8(24/78)	70(7/10)	88.2(60/68)	18
2	Spesis	348	18.1(63/348)	37.9(169/348) ^a^	48.6(169/348)	92.9(169/182)	62.7(104/166)	4
3	Community-acquired pneumonia (CAP)	15	6.7(1/15)	46.7(7/15) ^b^	86.7(13/15)	-	-	42
4	Pediatric infections	100	23.0(23/100)	52.0(52/100) ^c^	70.0(70/100)	91.8(56/61)	64.1(25/39)	102
5	iInvasive mycobacterium	10	50.0(5/10)	-	90.0(9/10)	-	-	53
6	Patients with fever of unknown origin, suspected respiratory infection, sepsis, suspected endocarditis or febrile neutropenia	82	19.5(16/82)	32.9(27/82)^ d^	61.0(50/82)	-	-	49
7	Pneumonia	18	-	-	66.7(12/18)	-	-	57
8	Pelapsed pediatric cancer patients with impending bloodstream infection (BSI)	47	-	-	-	83(15/18)	82(27/33)	26

Note: Other methods are microbiological tests including:** (a)** cultures, serology and nucleic acid testing; **(b)** standard culture and PCR based methods; **(c)** culture, PCR, morphology, serological test, etc.;** (d)** blood culture, tissue bacterial culture, viral PCR, etc.

## Abbreviations

mNGS: Metagenomic next-generation sequencing
mcfDNA: microbial cell free DNA
CSF: cerebrospinal fluid
PCR: polymerase chain reaction
LPS: lipopolysaccharide
ROS: reactive oxygen species
NETs: neutrophil extracellular traps
TB: tuberculosis
BSI: bloodstream infection
MRSA: Methicillin-resistant Staphylococcus aureus
AFB: acid-fast bacilli
BCG: Bacille Calmette-Guérin
IFD: Invasive Fungal Infections
GVHD: graft-versus-host disease
PCP: Pneumocystis pneumonia
VRE: vancomycin resistant Enterococci
AST: antimicrobial susceptibility testing
ARG: antibiotic resistance gene
SOP: standard operating procedure
EBV: Epstein-Barr Virus
ssDNA: single-stranded DNA
dsDNA: double-stranded DNA
gDNA: genomic DNA
HIV: human immunodeficiency virus
HCV: hepatitis C virus
RSV: respiratory syncytial virus
cfRNA: cell free RNA
